# Supplementary material for: Pirfenidone Alleviates Inflammation and Fibrosis of Acute Respiratory Distress Syndrome by Modulating the Transforming Growth Factor-β/Smad Signaling Pathway
Source: Int J Mol Sci. 2024 Jul 23;25(15):8014. doi: 10.3390/ijms25158014 (PMC11311955; doi:10.3390/ijms25158014)
Supplement: Supplementary file 1 [file ijms-25-08014-s001.zip › ijms-3054832-supplementary.pdf]

1. **Cell differ-counting of BALF**

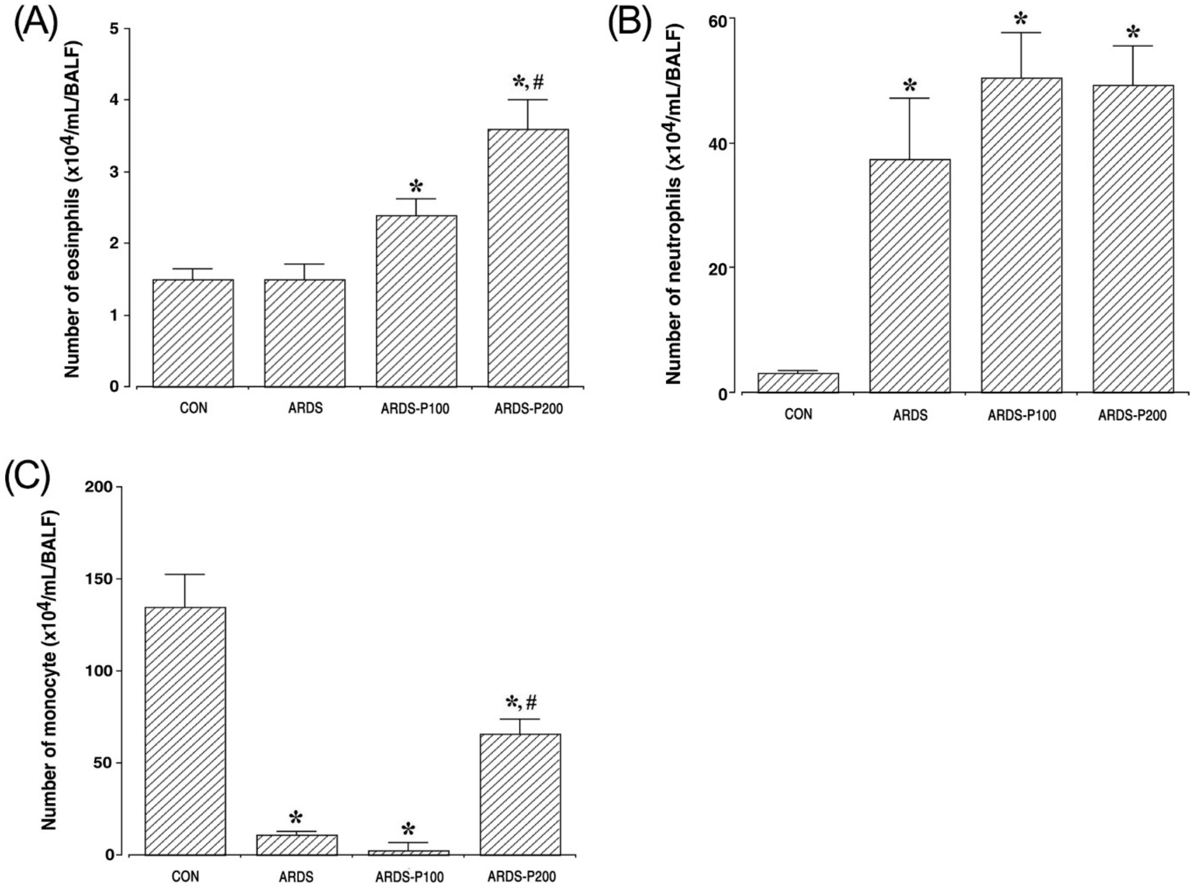

Supplementary Figure S1. Cell differ-counting of bronchoalveolar lavage fluid. * shows $P < 0.05$ compared to the control group. # shows $P < 0.05$ compared to the ARDS-inducing group. CON, control group; ARDS, acute respiratory distress syndrome-inducing group; ARDS-P100, acute respiratory distress syndrome-inducing and 100 mg/kg pirfenidone-treating group; ARDS-P200, acute respiratory distress syndrome-inducing and 200 mg/kg pirfenidone-treating group.
